# Vergleich der präklinischen Analgesiequalität von Notarzt und Notfallsanitäter anhand traumatologischer Patienten

**DOI:** 10.1007/s00101-024-01447-9

**Published:** 2024-08-06

**Authors:** Julian Thomas, Stefan Kleinschmidt, Philipp Mörsdorf, David Conrad, Ulrich Berwanger, Werner Armbruster

**Affiliations:** 1 Klinik für Anästhesie, Intensivmedizin und Schmerztherapie, Universitätsklinikum des Saarlandes, Gebäude 57, 66421 Homburg, Deutschland; 2 Klinik für Unfall‑, Hand-& Wiederherstellungschirurgie, Universitätsklinikum des Saarlandes, Gebäude 57, 66421 Homburg, Deutschland; 3 Klinik für Anästhesiologie, operative Intensivmedizin und Schmerztherapie, Marienhaus Klinikum St. Elisabeth, Kapuzinerstraße 4, 66740 Saarlouis, Deutschland

**Keywords:** Notfallsanitäter, Notarzt, Notfallmedizin, Präklinische Analgesie, Schmerztherapie, Emergency paramedic, Emergency medical service physician, Emergency medicine, Prehospital analgesia, Pain management

## Abstract

**Hintergrund:**

Die präklinische Schmerztherapie ist ein wesentlicher Bestandteil der Notfallmedizin. Mit Einführung des Berufsbildes des Notfallsanitäters wurden nicht ärztlichen Rettungskräften erweiterte medizinische Kompetenzen zuerkannt. Diese Studie evaluiert die Qualität der Analgesie durch Notfallsanitäter im Vergleich zu Notärzten anhand traumatologischer Patienten.

**Material und Methoden:**

Erfasst wurden Patienten mit Verletzungen, die nach einer präklinisch durchgeführten Analgetikagabe, durch Notärzte oder Notfallsanitäter, in der zentralen Notaufnahme des Universitätsklinikums des Saarlandes aufgenommen wurden. In einem Erfassungsbogen wurden sowohl aus dem Notfalleinsatzprotokoll als auch initial in der Sichtung/Notaufnahme erhobene Daten und Messwerte erfasst. Die deskriptive Auswertung erfolgte anhand statistischer Standardverfahren. Eingeschlossen wurden 207 komplette Datensätze. Als primärer Endpunkt wurde die Reduktion der Schmerzen bei Übergabe in der zentralen Notaufnahme festgelegt.

**Ergebnisse:**

Beide Berufsgruppen erreichen eine signifikante Reduktion der Schmerzen und erfüllen das Ziel einer effektiven Schmerztherapie (Schmerzdifferenz: Notarzt 5,5 ± 2,0/Notfallsanitäter 4,4 ± 2,1/*p* < 0,001). Notärzte erzielten eine höhere Reduktion der NRS-Werte und verabreichten häufiger Sauerstoff. Unterschiede zeigten sich v. a. im Spektrum der verwendeten Pharmaka.

**Schlussfolgerung:**

Bei korrekter Indikationsstellung und Beachtung der Handlungsanweisungen können Notfallsanitäter eine effektive Analgosedierung durchführen. Hinsichtlich Sicherheit und Qualität lässt sich die Therapie mit der eines Notarztes vergleichen.

## Abstract (en)

**Background:**

Adequate prehospital pain management is a critical component of emergency medical services. With the introduction of the paramedic profession and the Paramedics Act in Germany, the basis for more extensive competencies of paramedics was established. In many emergency medical service areas it is thus possible for paramedics to perform analgesia and sedation with esketamine/midazolam according to pre-established instructions and/or standard operating procedures. This study assessed the quality of analgesia administered to trauma patients by paramedics compared to emergency medical service physicians.

**Material and methods:**

The study included trauma patients who received prehospital administration of analgesia by either emergency medical service physicians or paramedics and were subsequently admitted to the central emergency department of the Saarland University Hospital. A standardized data collection form was used to collect information from the emergency service protocol and initial emergency department assessment. The evaluation employed descriptive statistical methods and a total of 207 completed records were analyzed.

**Results:**

Both professional groups achieved significant pain reduction and fulfilled the criteria for effective pain management (pain reduction: emergency medical service physicians 5.5 ± 2.0/paramedic 4.4 ± 2.1, *p* < 0.001). Emergency medical service physicians, however, more frequently attained a higher reduction in numerical rating scale scores and administered oxygen. Notable differences were observed in the range of medications used and the dosages.

**Conclusion:**

This study could show that prehospital analgesia is comparable between emergency medical service physicians and paramedics in terms of effectiveness for trauma patients if the indications are correctly set, while observing pre-existing instructions. With their competencies paramedics are able to perform an effective and safe analgesic treatment within the framework of preformulated procedural instructions, which can be equal to that of an emergency medical service physician.

Mit der Einführung des Berufsbildes des Notfallsanitäters und dem Gesetz über den Beruf des Notfallsanitäters [2] wurden vom Gesetzgeber die Grundlagen für weitreichende medizinische Kompetenzen geschaffen. So ist es in vielen Rettungsdienstbereichen möglich, dass Notfallsanitäter nach vorgefertigten Handlungsanweisungen eine Analgosedierung mit Esketamin/Midazolam selbstständig durchführen. Die vorliegende Studie untersucht anhand traumatologischer Patienten, ob sich die präklinische Analgesie zwischen Notfallsanitäter und Notarzt in der Qualität sowie in den Patientengruppen unterscheidet.

## Hintergrund

In der prähospitalen Notfallmedizin sind Schmerzen ein häufiger Alarmierungsgrund für den Rettungsdienst. Angaben zur präklinischen Prävalenz von Schmerzen variieren stark, und Studienergebnissen zufolge schildern etwa 20 % der Patienten im Rettungsdienst Schmerzen [1, 5]. Die größte Gruppe repräsentieren hierbei Patienten mit Traumata. Ihr Anteil beträgt in Deutschland etwa 18 % der Rettungsdiensteinsätze [1], und sie machen etwa 50 % der Rettungsdiensteinsätze, bei denen Schmerzen geäußert werden, aus. Die restlichen 50 % unterteilen sich in Thoraxschmerzen bei kardiopulmonalen Erkrankungen, akute abdominelle Schmerzen sowie sonstige akute Schmerzsyndrome [11, 12, 15]. Die Behandlung von Schmerzen stellt ein zentrales Element der Akutversorgung dar. Sie ist nicht nur aus ethischen, sondern auch aus medizinischen Gründen indiziert, um negative Auswirkungen auf den Patienten zu verhindern [4]. Bis in die jüngste Vergangenheit war die Schmerztherapie in Deutschland eine rein ärztliche Aufgabe. Dies führte häufig zur Primäralarmierung eines arztbesetzten Rettungsmittels oder zu einer Nachalarmierung von Notärzten an die Einsatzstelle. Alternativ wurden Patienten ohne ausreichende analgetische Therapie durch den Rettungsdienst transportiert. Seit der Einführung des Berufsbildes des Notfallsanitäters wird die ärztliche Aufgabe auch von diesen übernommen. Dennoch gibt es noch keine bundeseinheitlichen Empfehlungen, sodass sich die Therapiemöglichkeiten der Notfallsanitäter regional stark unterscheiden. Dem nicht ärztlichen Personal stehen häufig nur wenige potente Analgetika zur Verfügung. Hochpotente Opiate und Opioide unterliegen weitgehend dem Betäubungsmittelgesetz. Das Betäubungsmittelgesetz stellt eine erhebliche Hürde für eine Bereitstellung von Opiaten und Opioiden zur Applikation durch Notfallsanitäter dar. Die Befugnisse der Notfallsanitäter im Saarland werden in der „Verfahrensanweisung ORG-02: Medizinische Kompetenz des Notfallsanitäters“ festgehalten [18]. Demnach ist eine Analgosedierung mit Esketamin und Midazolam, entsprechend einer vorgefertigten Handlungsempfehlung, freigegeben.

In unserer Studie wurde untersucht, ob sich die präklinische Analgesie zwischen Notärzten und Notfallsanitätern, sowohl in der Qualität, in der Art und Weise als auch in den Patientengruppen unterscheidet. Um einen direkten Vergleich der Analgesiequalität, unabhängig der Substanzklasse des Analgetikums, zu ermöglichen, wurde eine Subgruppenanalyse durchgeführt. Dabei wurden nur Patienten berücksichtigt, die sowohl von Notarzt als auch von Notfallsanitäter ausschließlich mit Esketamin/Midazolam behandelt wurden.

### Material und Methoden

Das Studienprotokoll wurde von der Ethikkommission der Ärztekammer des Saarlandes genehmigt (Registriernummer 10/20 mit Schreiben vom 08.01.2020).

### Studiendesign

Es handelt sich um eine prospektive Beobachtungsstudie. Erfasst wurden traumatologische Patienten mit Einfach- oder Mehrfachverletzungen, die nach einer präklinischen Analgetikagabe in der zentralen Notaufnahme des Universitätsklinikums des Saarlandes aufgenommen wurden. Ausgeschlossen wurden Patienten mit vitaler Bedrohung, Patienten unter 18 Jahren, unvollständig dokumentierte Fälle und Patienten, die von einem Notfallsanitäter und zusätzlich von einem nachgeforderten Notarzt analgetisch behandelt wurden. Bei Übernahme in der Notaufnahme wurde ein Erfassungsbogen angelegt. Erfasst wurden die Vitalparameter, verabreichte Pharmaka, behandelnde Personen und die Verletzungsdiagnose. Hinsichtlich der Schmerzintensität wurden die Patienten zu 2 Zeitpunkten befragt. Es wurden der initiale Schmerz nach Trauma sowie der aktuelle Schmerz bei Übergabe dokumentiert. Um die Schmerzintensität zu erfassen, wurde die numerische Ratingskala (NRS) benutzt. Es wurden sowohl aus dem Notfalleinsatzprotokoll als auch initial in der Sichtung/Notaufnahme erhobene Daten und Messwerte erfasst. Zusätzlich erfolgte eine Nachverfolgung mithilfe der SAP-Datenbank des Universitätsklinikums des Saarlandes. Um einen Vergleich hinsichtlich der Verletzungsschwere zu ermöglichen, wurden die Patienten nach Durchlaufen der Diagnostik mithilfe der Abbreviated Injury Scale (AIS) und des Injury Severity Score (ISS) klassifiziert. Es wurden 207 komplette Datensätze erfasst. Von diesen wurden 82 Patienten von einem Notfallsanitäter und 125 Patienten von einem Notarzt versorgt. In die Subgruppenanalyse wurden 121 Patienten eingeschlossen. Davon wurden 53 Personen von einem Notarzt und 68 Personen von einem Notfallsanitäter behandelt. Der Erfassungszeitraum lag zwischen Mai 2020 und Juli 2021.

### Statistische Analyse

Vor Beginn der Datenerfassung erfolgte die Durchführung einer Fallzahlberechnung mithilfe von G*Power (Statistical Power Analyses Windows – Version 3.1.9.7). Dabei wurde eine mittlere Effektstärke von d = 0,5 gewählt. Der Fehler 1. Art betrug α = 0,05 und die Power 0,86. Es wurde eine Gruppengröße von jeweils 64 Patienten errechnet. Die Gesamtzahl sollte demnach mindestens 140 Studienteilnehmer betragen (Drop-out-Rate 10 %). Die Daten wurden anonymisiert und in einer Excel-Tabelle (Microsoft Excel für Microsoft 365 MSO-Version 2209) gesammelt. Anschließend wurden die Daten in IBM SPSS Version 27 (Statistical Package for the Social Sciences) übernommen. Die deskriptive Auswertung erfolgte anhand statistischer Standardverfahren.

Die Normalverteilungsannahmen wurden mit dem Kolmogorov-Smirnov-Test überprüft. Bei unabhängigen Stichproben und fehlender Normalverteilung der Daten wurde der Mann-Whitney-U-Test verwendet, um die Nullhypothese zu überprüfen. Zur Überprüfung auf signifikante Unterschiede der Patienteneigenschaften wurden Chi-Quadrat-Tests verwendet. Um die Schmerzreduktion in den beiden Gruppen zu überprüfen, wurde bei fehlender Normalverteilung der Vorzeichen-Test für verbundene Stichproben angewandt. Das Signifikanzniveau war für alle Tests auf *p* ≤ 0,05 festgelegt. Stellte sich ein statistisch signifikanter Unterschied zwischen den Gruppen dar, wurden die entsprechenden *p*-Werte angegeben. Um die Power in der Subgruppenanalyse zu bestätigen, wurde eine zweite Analyse mit G*Power durchgeführt. Bei einer mittleren Effektstärke von d = 0,5, einem Fehler 1. Art α = 0,05, zeigte sich für unsere Stichprobengröße eine Power von 0,84.

## Ergebnisse

### Demografische Daten

Hinsichtlich der demografischen Daten zeigen sich keine Unterschiede zwischen den beiden Gruppen (Tab. 1).

**Tab. 1:** Demografische Daten

Gruppe	Geschlecht				Alter [Jahre]	Gewicht [kg]
	Männlich		Weiblich			
	Anzahl [*n*]	Prozent [%]	Anzahl [*n*]	Prozent [%]		
Notarzt	57	45,6	68	54,4	58,8 ± 22,2	75,6 ± 14,4
Notfallsanitäter	30	36,6	52	63,4	61,5 ± 22,7	75,2 ± 16,4

### Vitalparameter

Bei den gemessenen Vitalparametern zeigen sich bei den initialen GCS-Werten, dem initial gemessenen Blutdruck sowie den bei Übergabe in der Notaufnahme festgestellten GCS-Werten und der Herzfrequenz signifikante Unterschiede. Dabei handelt es sich jedoch um statistische Unterschiede. Eine klinische Relevanz war nicht vorhanden (Tab. 2).

**Tab. 2:** Vitalparameter initial und bei Übergabe

Gruppe	GCS			RR [mm Hg]		HF [bpm]		S_p_O_2_ [%]	
	Initial	Übergabe	Differenz	Initial	Übergabe	Initial	Übergabe	Initial	Übergabe
Notarzt	14,9 ± 0,6	14,6 ± 0,9	0,3 ± 0,8	139,9 ± 25,3	136,6 ± 18,4	87,1 ± 15,5	83,1 ± 12,9	97,0 ± 2,5	97,9 ± 1,8
Notfallsanitäter	15,0 ± 0,0	14,7 ± 1,0	0,3 ± 1,0	130,6 ± 26,0	131,2 ± 20,0	85,2 ± 15,5	79,4 ± 11,5	97,1 ± 2,2	97,7 ± 1,7
*p*-Wert	0,030	0,024	–	0,020	–	–	0,022	–	–

### Schmerzintensität

**Abb. 1:**
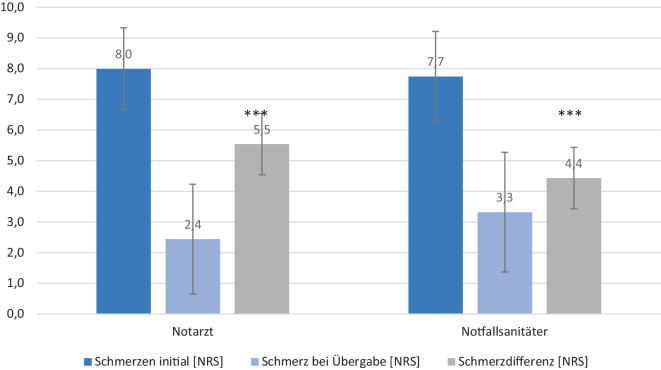
Schmerz initial und bei Übergabe/Schmerzreduktion (***= *p* < 0,001)

Vergleicht man die Schmerzintensitäten auf der NRS zwischen beiden Gruppen stellt man fest, dass es keinen statistisch signifikanten Unterschied der initialen Schmerzintensität gibt (Notarzt 8,0 ± 1,3/Notfallsanitäter 7,7 ± 1,5). Zum Zeitpunkt der Übergabe in der zentralen Notaufnahme erreichen beide Gruppen eine signifikante Reduktion der Schmerzen (Notarzt 2,4 ± 1,8/Notfallsanitäter 3,3 ± 2,0/*p* < 0,001). Vergleicht man die festgestellten NRS-Werten bei Übergabe in der zentralen Notaufnahme, stellt sich in der notärztlich versorgten Patientengruppe eine signifikant höhere Reduktion der Schmerzen durch die medikamentöse Intervention dar (Schmerzdifferenz: Notarzt 5,5 ± 2,0/Notfallsanitäter 4,4 ± 2,1/*p* < 0,001). (Abb. 1).

### Verletzungsschwere

Die Verletzungsschwere (erfasst im ISS) ist in der nicht ärztlich versorgten Patientengruppe tendenziell etwas höher. Beide Gruppen befinden sich jedoch im Bereich von leichten bis mittelschweren Verletzungen (ISS < 16). Hinsichtlich der Hospitalisierungsdauer zeigen sich keine Unterschiede (Tab. 3). Der im Mittel etwas höhere ISS in der Gruppe der Notfallsanitäter hatte demnach keinen Einfluss auf die Dauer des Krankenhausaufenthaltes.

**Tab. 3:** Verletzungsschwere ISS und Hospitalisierungsdauer

Gruppe	ISS	Hospitalisierungsdauer [Tage]
Notarzt	3,92 ± 3,09	7,12 ± 8,94
Notfallsanitäter	4,85 ± 3,12	6,49 ± 7,38
*p*-Wert	0,028	

### Sauerstoffgabe

Betrachtet man die Häufigkeit einer Sauerstoffgabe während der präklinischen Phase, zeigt sich, dass Notärzte signifikant häufiger Sauerstoff verabreicht haben (Tab. 4).

**Tab. 4:** Häufigkeit der Sauerstoffgabe

Gruppe	Sauerstoffgabe			
	Erfolgt		Nicht erfolgt	
	Anzahl [*n*]	Prozent [%]	Anzahl [*n*]	Prozent [%]
Notarzt	38	30	87	70
Notfallsanitäter	14	17	68	83
*p*-Wert	0,034			

### Pharmaka

**Abb. 2:**
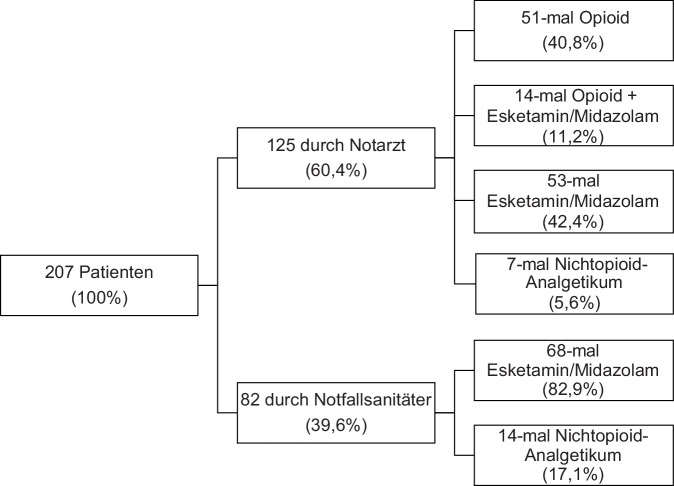
Studienpopulation mit eingesetzten Medikamenten

Unterschiede zeigen sich auch im Spektrum der eingesetzten Pharmaka (Abb. 2). Von Notärzten wurde in 40,8 % der Fälle ein Opioid zur analgetischen Therapie eingesetzt. In 11,2 % der Fälle wurde ein Opioid mit Esketamin/Midazolam kombiniert. Die Nichtopioidanalgetika (Paracetamol oder Metamizol in körpergewichtsadaptierten Standarddosierungen) kamen bei 5,6 % der Patienten zum Einsatz. Die restlichen 42,4 % erhielten Esketamin/Midazolam. Aufgrund der im Saarland geltenden Vorgaben stehen Notfallsanitätern keine Opioidanalgetika zur Verfügung. Notfallsanitäter benutzten in 82,9 % Esketamin/Midazolam. Bei 17,1 % der dokumentierten Fälle wurde Paracetamol verabreicht (Abb. 2).

Die Ergebnisse der Subgruppenanalyse sind mit der Hauptanalyse weitgehend deckungsgleich. Jedoch zeigen sich signifikante Unterschiede hinsichtlich der Medikamentendosierungen. In der notärztlich versorgten Patientengruppe wurde signifikant mehr Esketamin und mehr Midazolam verabreicht (Tab. 5).

**Tab. 5:** Dosierungen von Esketamin und Midazolam

Gruppe	Esketamin [mg/kgKG]	Midazolam [mg/kgKG]
Notarzt	0,34 ± 0,19	0,03 ± 0,02
Notfallsanitäter	0,20 ± 0,11	0,02 ± 0,01
*p*-Wert	*p* < 0,001	*p* < 0,001

## Diskussion

Die anfängliche Schmerzintensität unterscheidet sich zwischen beiden Gruppen nicht, und die Intensität der Schmerzen ist bei allen Probanden initial als stark anzusehen. Die hohe Schmerzintensität lässt sich dadurch erklären, dass nur Patienten in die Untersuchung aufgenommen wurden, die auch tatsächlich eine medikamentöse Schmerztherapie erhalten haben. Wie eine andere Studie bereits zeigt, werden Patienten mit starken Schmerzen häufiger medikamentös analgetisch therapiert als solche mit mittelstarken oder nur leichten Schmerzen [16]. In beiden Gruppen gaben die Betroffenen bei Übergabe in der zentralen Notaufnahme deutlich geringere Schmerzen auf der NRS an. In beiden Behandlungsgruppen wurde eine signifikante Reduktion der Schmerzintensität erreicht. Diese Schmerzlinderung wird von den Patienten als Maß der präklinischen Versorgungsqualität herangezogen, um den Erfolg der Therapie zu beurteilen. Das Ziel einer effektiven Schmerztherapie (NRS < 4 oder eine Schmerzreduktion um mindestens 3 Punkte) [13] haben demnach beide Gruppen erreicht. Somit können wir, wie bereits in anderen Studien [7, 8] gezeigt wird, nachweisen, dass eine effektive Schmerztherapie durch nicht ärztliches Rettungsfachpersonal durchgeführt werden kann. Bei Übergabe zeigte sich die Schmerzintensität in der notärztlich versorgten Patientengruppe signifikant niedriger als im Patientenkollektiv der Notfallsanitäter. Vergleicht man die Schmerzdifferenz zwischen den beiden Gruppen, zeigt sich, dass durch die notärztliche Therapie eine höhere Schmerzreduktion erreicht wird. Der Unterschied zwischen den beiden Gruppen ist statistisch signifikant (*p* < 0,001). Dies führen wir darauf zurück, dass dem ärztlichen Personal ein breiteres Spektrum an Analgetika zur Verfügung steht, u. a. hochpotente Opioidanalgetika. Zudem sind die Dosierungen der Nichtopioidanalgetika für Notfallsanitäter durch Verfahrensanweisungen begrenzt. Durch Notärzte können demnach höhere Dosierungen der Medikamente gegeben werden. Auch eine Kombination verschiedener Analgetika (beispielsweise Opioid + Nichtopioidanalgetikum) ist dem Notarzt vorbehalten. Betrachtet man die Vitalparameter initial und bei Übergabe, zeigt sich, dass durch einen Notfallsanitäter eine mindestens gleichwertige Analgesie, hinsichtlich der Patientensicherheit (im Sinne einer Vermeidung einer Hypoxie), durchgeführt wurde. Bezüglich der Verletzungsschwere zeigt sich für die Studienpopulation, dass in der nicht ärztlich versorgten Patientengruppe der ISS als Maß für die Verletzungsschwere tendenziell sogar etwas höher lag (*p* = 0,028). Beide Gruppen befinden sich hinsichtlich der Verletzungsschwere jedoch im Bereich von leichten bis mittelschweren Verletzungen. Dies zeigt, dass die Einschlusskriterien für die Patientenselektion gut gewählt wurden. Zudem hatte der leicht höhere ISS der nicht ärztlich versorgten Patientengruppe keinen Einfluss auf die initiale Schmerzintensität oder die Hospitalisierungsdauer, da hier in beiden Gruppen keine signifikanten Unterschiede feststellbar sind.

Bezüglich der initial und bei Übergabe gemessenen Sauerstoffsättigung zeigen sich keine Unterschiede zwischen den Gruppen. Notärzte verabreichten jedoch signifikant häufiger Sauerstoff als Notfallsanitäter (*p* = 0,034). Daraus könnte man schlussfolgern, dass es während der notärztlichen Versorgung häufiger zu einem Abfall der Sauerstoffsättigung kam, der jedoch mit einer Sauerstoffinsufflation behandelt werden konnte. Wir erklären dies durch die Unterschiede bezüglich der verabreichten Medikamente und der einhergehenden Medikamentenwirkungen. Speziell bei Analgetika aus der Gruppe der Opioide ist eine atemdepressive Wirkung bekannt [17]. Aber auch bei Midazolam und Esketamin konnte eine atemdepressive Wirkung nachgewiesen werden [3, 10]. Zudem wäre in der notärztlich versorgten Patientengruppe der bewusste Einsatz höherer Medikamentendosierungen denkbar, um die Patienten von der Situation abzuschirmen und damit einhergehend ein häufigerer Bedarf einer Sauerstoffinsufflation.

Wie bereits beschrieben, hatten die Betroffenen initial starke Schmerzen angegeben. Daher ist es nicht verwunderlich, dass überwiegend Analgetika mit hoher analgetischer Potenz (Opioide, Esketamin) eingesetzt wurden und nur in wenigen Fällen die weniger analgetisch wirksamen Nichtopioidanalgetika Verwendung fanden. Wie eine große Metaanalyse bereits 2017 zeigte, sind sowohl die Opioide Fentanyl und Morphin als auch Ketamin als potente Analgetika beim spontanatmenden Traumapatienten geeignet, und es konnte keine Überlegenheit für eine der drei Substanzen gezeigt werden [9]. Auch in der von Notfallsanitätern behandelten Patientengruppe wurden vermehrt Esketamin/Midazolam eingesetzt. Im Saarland ist den Notfallsanitätern laut Verfahrensanweisung als Analgetika lediglich Paracetamol als Nichtopioidanalgetikum und Esketamin/Midazolam freigegeben. Auch hier ist aufgrund der hohen Schmerzintensität nachvollziehbar, dass vermehrt das analgetisch potentere Medikament zum Einsatz kam.

In unserer Subgruppenanalyse können wir die Unterschiede hinsichtlich der Medikamentendosierungen zeigen. Die Patientengruppe, die von einem Notarzt behandelt wurde, erhielt signifikant höhere Dosen Esketamin und höhere Dosen Midazolam. Wir führen die stärkere Schmerzreduktion in der notärztlich versorgten Patientenklientel auf die unterschiedlichen Dosierungen von Esketamin und Midazolam zurück. Hinsichtlich der Vitalparameter zeigten sich allenfalls statistische Unterschiede ohne klinische Relevanz. Aufgrund dieser Tatsache lässt sich die Frage diskutieren, ob die in SOP festgelegten Dosierungsgrenzen, bezüglich Esketamin oder Midazolam, angepasst werden sollten. Dann könnte man untersuchen, ob ein Notfallsanitäter mit höheren Dosierungen eine gleichwertige Reduktion der NRS-Werte bei gleichbleibender Patientensicherheit erreichen kann, wie ein Notarzt, oder ob evtl. noch andere Faktoren eine Rolle spielen.

Deutschlandweit gibt es in weiteren Rettungsdienstbereichen verschiedene Konzepte bezüglich präklinischer Analgesie durch Rettungsfachpersonal. Durch Gnirke et al. wurde 2019 eine Vergleichsstudie zwischen Telenotarzt- und Callback-Verfahren hinsichtlich Anwendungssicherheit, Wirksamkeit und Verträglichkeit durchgeführt. Auch dort konnte gezeigt werden, dass die Applikation von Analgetika durch Rettungsdienstpersonal sicher anwendbar ist. Durch telemedizinische oder telefonisch ärztliche Konsultation wurde eine hochwertige Patientenversorgung, auch bei nicht traumatologisch bedingten Schmerzzuständen sichergestellt [6]. Zu einem ähnlichen Ergebnis kamen Lenssen et al., hier wurde die Analgesie sowohl bei Traumaschmerz als auch bei Nontraumaschmerz nach festen Algorithmen durch Notfallsanitätern unterstützt durch einen Telenotarzt durchgeführt. Im Vergleich zu einer Analgesie durch Notärzte vor Ort konnte in beiden Gruppen ein signifikanter Unterschied im Rückgang der Schmerzstärke festgestellt werden. Die beiden Gruppen unterschieden sich in der Qualität der Analgesie nicht [14]. Im Saarland sind derartige Konzepte bisher jedoch nicht etabliert.

## Fazit

- Wir konnten mit unserer Untersuchung zeigen, dass bei korrekter Indikationsstellung und Beachtung der Handlungsanweisungen die präklinische Analgesie zwischen Notarzt und Notfallsanitäter bei traumatologischen Patienten hinsichtlich der Effektivität vergleichbar ist.
- Mit eigenen Kompetenzen können Notfallsanitäter eine effektive und sichere Analgosedierung, die sich mit jener eines Notarztes messen lässt, im Rahmen vorgefertigter Verfahrensanweisungen durchführen.
- Unsere Untersuchung betrachtete jedoch nur traumatologische Patienten, da im Saarland nur für dieses Kollektiv eine Therapie mit Esketamin/Midazolam durch Notfallsanitäter freigegeben ist.
- Bei nichttraumatisch bedingten Schmerzzuständen gibt es also in vielen Rettungsdienstbereichen immer noch Verbesserungsmöglichkeiten, da dort eine analgetische Therapie häufig noch dem Notarzt vorbehalten ist.
